# Specific Cytokine Profiles Predict the Severity of Influenza A Pneumonia: A Prospectively Multicenter Pilot Study

**DOI:** 10.1155/2021/9533044

**Published:** 2021-10-13

**Authors:** Yu Xie, Yan Yu, Lili Zhao, Pu Ning, Qiongzhen Luo, Ying Zhang, Lu Yin, Yali Zheng, Zhancheng Gao

**Affiliations:** ^1^Department of Respiratory & Critical Care Medicine, Peking University People's Hospital, No. 11, Xizhimen South Street, Xicheng District, Beijing, China; ^2^Department of Respiratory and Critical Care Medicine, The Second Affiliated Hospital of Xi'an Jiaotong University, 157 West Fifth Road, Xi'an, Shaanxi, China; ^3^Department of Respiratory and Critical Care Medicine, Beijing Tsinghua Changgung Hospital, 168 Litang Road, Beijing, China; ^4^Department of Respiratory, Critical Care and Sleep Medicine, Xiang'an Hospital of Xiamen University, 2000 Xiang'an East Road, Xiamen, Fujian, China

## Abstract

**Purpose:**

Studying the cytokine profiles in influenza A pneumonia could be helpful to better understand the pathogenesis of the disease and predict its prognosis. *Patients and Methods*. Patients with influenza A pneumonia (including 2009H1N1, H1N1, H3N1, and H7N1) hospitalized in six hospitals from January 2017 to October 2018 were enrolled (ClinicalTrials.gov ID, NCT03093220). Sputum samples were collected within 24 hours after admission and subsequently analyzed for cytokine profiles using a Luminex assay.

**Results:**

A total of 35 patients with influenza A pneumonia were included in the study. The levels of IL-6, IFN-*γ*, and IL-2 were increased in patients with severe influenza A pneumonia (n =10) (*P* = 0.002, 0.009, and 0.008, respectively), while those of IL-5, IL-25, IL-17A, and IL-22 were decreased compared to patients with nonsevere pneumonia (*P* = 0.0001, 0.009, 0.0001, and 0.006, respectively). The levels of IL-2 and IL-6 in the nonsurvivors (*n* = 5) were significantly higher than those in the survivors (*P* = 0.043 and 0.0001, respectively), while the levels of IL-5, IL-17A, and IL-22 were significantly lower (*P* = 0.001, 0.012, and 0.043, respectively). The IL-4/IL-17A ratio has the potential to be a good predictor (AUC = 0.94, *P* < 0.05, sensitivity = 88.89%, specificity = 92.31%) and an independent risk factor (OR, 95% CI: 3.772, 1.188-11.975; *P* < 0.05) for intermittent positive pressure ventilation (n = 9).

**Conclusion:**

Significant dysregulation of cytokine profiles can be observed in patients with severe influenza A pneumonia.

## 1. Introduction

Influenza A viral pneumonia is a disease with high morbidity and mortality. According to the World Health Organization (WHO), the 2009 influenza A pandemic affected 213 countries and caused more than 16,000 deaths [1]. Subsequent surveillance data from the Centers for Disease Control and Prevention (CDC) showed that the number of patients reporting influenza-like symptoms continues to rise each year during the flu season. The majority of laboratory-diagnosed influenza-related hospitalizations are among people older than 65 years of age [2]. A study has shown that among people who die from influenza pneumonia, the proportion of patients aged 65 years or older can be as high as 96% [3]. With the increasing age of the population in China, influenza will continue to cause a great social and economic burden. Early and effective warning of the disease progression helps to improve clinical management while avoiding wasting medical resources.

It is widely believed that inflammation is part of the pathogenesis of influenza [4]. Higher levels of proinflammatory cytokines and cytokine dysregulation were found in patients with H1N1 influenza [5]. Excessive release of proinflammatory cytokines, including tumor necrosis factors (TNFs), interleukins (ILs), interferons (IFNs), and chemokines, follows infections are called “cytokine storms” and are associated with widespread tissue damage and complications due to severe influenza [6]. In most cases, the damage caused by acute inflammation can be completely recovered through the repair process, but more severe pathological changes can occur in serious inflammation related to cytokine storms, including diffuse alveolar damage, hyaline membrane formation, pathological damage, and even multiple organ dysfunctions [7]. Previous studies revealed that excessive early cytokine responses strongly predict poor medical outcomes, and the patients with good prognosis had markedly lower cytokine storm profiles than those who died from the infection [8].

Previous studies have suggested that immunomodulatory therapy can improve prognosis in influenza treatment, regardless of if they are combined with antiviral drugs [4, 7]. Immunoregulatory treatments include corticosteroids, sphingosine 1-phosphate receptor agonists, peroxidase, proliferation-activating receptor agonists [8], cyclooxygenase- (COX-) 2 inhibitors [7, 9, 10], antioxidants [11], intravenous immunoglobulin therapy, antitumor necrosis factor therapy [12], statins, abiddo, and herbs [13]. However, most of the studies were from a small sample population, and the outcome reports were heterogeneous [14, 15]; so, more precise indicators were needed to identify populations likely to benefit from immunoregulatory therapy.

This study is aimed at determining the airway Th cell cytokine profiles in patients with influenza A pneumonia and the relationships between cytokine levels and prognosis.

## 2. Materials and Methods

### 2.1. Study Populations

Patients diagnosed with community-acquired pneumonia (CAP) and hospitalized in six hospitals (Peking University People's Hospital, Fujian Provincial Hospital, West China Hospital of Sichuan University, Shanghai Pulmonary Hospital, The Second Hospital of Jilin University, and Tibet Autonomous Region People's Hospital) from January 2017 to October 2018 were successively included (ClinicalTrials.gov ID, NCT03093220).

According to the definition of community-acquired pneumonia and severe pneumonia, patients who meet the criteria were included strictly. The treatment of each patient during hospitalization was rigorously carried out in accordance with the guidelines for pneumonia [16]. The inclusion criteria of our study were as follows: [1] patients who met the diagnostic criteria of CAP and [2] patients whose sputum samples were positive for influenza A (including 2009H1N1, H1N1, H3N1, and H7N1). The diagnostic criteria of CAP and severe pneumonia, the exclusion criteria, and detailed information on pathogen detection are described in the (available here) Supplementary Materials.

**Figure 1:**
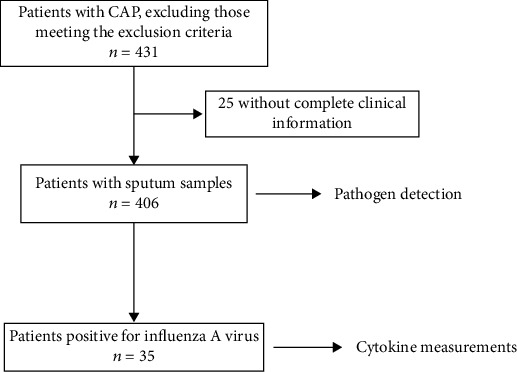
Flow diagram of the study. Abbreviations: CAP: community-acquired pneumonia.

A standard electronic medical spreadsheet was used to collect clinical information from the patients while in the hospital. Patients all gave written informed consent before their participation in the study. The Ethics Committee of Peking University People's Hospital approved the study. This trial was conducted in accordance with the Declaration of Helsinki. A flow diagram of the study is shown in Figure 1.

### 2.2. Collection of Sputum Specimens

Spontaneous sputum samples were collected aseptically within 24 hours after admission for every patient. Microscopes were used to measure sputum quality prior to processing. Specimens were selected if <10 squamous epithelial cells, and > 25 leukocytes were present per low-power field (×100 objective). To ensure consistency and standardization of experimental work, all specimens were transported promptly to the laboratory in Peking University People's Hospital with dry ice in temperature-controlled containers, checked for integrity upon arrival, and stored in a temperature-appropriate environment in a timely manner. Sputum samples were discharged into sterile cups and then incubated with 1× volume of 0.1% dithiothreitol (DTT) solution at 37°C for 30 minutes. Subsequently, an equal volume of sterile saline was mixed with the samples (compared to the DTT solution), and sample was shaken for 5 minutes and then centrifuged at 12000 rpm for 10 minutes at room temperature [17, 18]. The supernatants were transferred to new centrifuge tubes and cryopreserved at -80°C along with the remaining precipitates. All of the DNA extraction, cytokine measurements, and detection of respiratory pathogens experiments were performed at Peking University People's Hospital, using a single standardized protocol (described detailly below).

### 2.3. Cytokine Measurements

For the sputum supernatants, a total of 12 T helper (Th) cytokines were evaluated with the Luminex Human Magnetic Assay Kit (LXSAHM-12; R&D Systems, Minneapolis, MN, USA) used for the quantification of the following cytokines: Th1 (TNF-*α*, IFN-*γ*, and IL-2), Th2 (IL-4, IL-5, and IL-25), Th17 (IL-6, IL-17A, IL-21, IL-22, and IL-23), and Treg (IL-10) cytokines, according to the manufacturer's instructions. Detailed information has been described in an article previously published [19].

### 2.4. Detection of Respiratory Pathogens

Microbial DNA was isolated from sputum sediments using a sputum microbial DNA extraction kit (CapitalBio Corporation, Beijing, China) as instructed by the manufacturer. Viral nucleic acids were isolated from the sputum supernatants using a QIAamp MinElute Virus Spin Kit (QIAGEN, Germany) as instructed by the manufacturer. Nucleic acids were detected for 76 kinds of common respiratory pathogens (35 kinds of bacteria, 3 kinds of atypical pathogens, 20 kinds of fungi, and 18 kinds of viruses) using Chips for Complicated Infection Detection (CCID; CapitalBio Corporation, Beijing, China) as instructed by the manufacturer. Detailed information and the procedure of nucleic acid extraction and pathogen detection are provided in the (available here) Supplementary Methods.

### 2.5. Statistical Analysis

Cytokines with ≥30% of samples under the lower limit of detection (LOD) were excluded from our analysis [20]. Values that were under the lower LOD were imputed by a value of one-half of the lower LOD [21]. Data are expressed as mean ± standard deviation (SD) or median values and interquartile ranges (IQRs). Mann–Whitney *U* and Kruskal-Wallis tests are used for nonnormally distributed variables while Student's *t*-test and ANOVA are used for normally distributed variables. Fisher's exact test and the chi-square test were used to compare the proportions of the groups. Receiver operating characteristic (ROC) analysis was performed to differentiate patients who received IPPV from those who did not. Binary logistic regression was used to determine the risk factors for IPPV. IBM SPSS Statistics 25.0 (IBM, 2017) and GraphPad Prism version 7.0 were used for statistical analysis.

## 3. Results

### 3.1. Characteristics of Patients

A total of 35 patients (23 men and 12 women) with influenza A pneumonia were included in the study, with a mean age of 64 ± 17 years. Among them, nine patients received intermittent positive pressure ventilation (IPPV), and five died. The patients were further grouped according to clinical characteristics into severe pneumonia (SP) and nonsevere pneumonia (NSP). The rate of patients with comorbidities was significantly higher in the NSP group; however, no significant difference was found between the two groups for any single comorbidity (COPD, bronchiectasis, type 2 diabetes, or cardiovascular disease), as shown in Table 1. Compared with patients in the NSP group, patients in the SP group had lower lymphocytes and PaO_2_/FiO_2_ and a higher neutrophil/lymphocyte ratio (NLR), C-reactive protein (CRP), and procalcitonin (PCT). Patients in the SP group had a significantly poorer prognosis, with a higher proportion of noninvasive positive pressure ventilation (NIPPV), IPPV, intensive care unit (ICU) admission, and death.

### 3.2. Cytokines and Pathogen Detection

Levels of Th cell cytokines in the sputum supernatant were detected. The median levels and percentages of measurements below the lower LOD are shown in (available here) Supplementary Table 1. IL-21, which had 80% measurements below the lower LOD, was excluded from further analysis in the study.

Among the 35 influenza A virus-positive patients we included, 26 patients were identified to specific subtypes. Among them, 10 were positive for 2009H1N1, and 15 were positive for H3N1, while only 1 was positive for H1N1 and no patient was positive for H7N1. We compared clinical characteristics and cytokine levels in patients who were positive for 2009H1N1 or H3N1. In general, patients positive for 2009H1N1 had decreased lymphocytes (0.8 × 10^9^/L and 1.4 × 10^9^/L, *P* = 0.024), elevated IL-2 levels (95.30 pg/ml and 58.57 pg/ml, *P* = 0.019) and decreased IL-5 levels (1.59 pg/ml and 4.21 pg/ml, *P* = 0.004) compared with patients positive for H3N1 (as shown in (available here) Supplementary Table 3 and Supplementary Table 4).

Pathogens other than influenza A virus were detected in 24 cases (68.6%). Among them, other respiratory viruses were detected in 13 cases (37.1%), and adenovirus was the most common virus detected (17.1%, 6/35), followed by influenza B virus, herpes simplex virus (HSV)-1, and cytomegalovirus, with the same detection rate of 5.7% (2/35). Bacteria were detected in 9 cases (25.7%), with the highest rate being that of *Streptococcus pneumoniae*, *Pseudomonas aeruginosa* , and *Staphylococcus haemolyticus*, with the same detection rate of 5.7% (2/35). Fungi were detected in 6 cases (17.1%), with the *Aspergillus genus* found in 5 cases (14.3%) and *Candida tropicalis* and *Candida krusei* in 1 case (2.9%). *Candida albicans* was detected in 14 cases (40.0%), and Epstein-Barr virus (EBV) was detected in 13 cases (37.1%). Considering that *Candida albicans* is a common oral colonization fungus, and we used sputum samples, we excluded *Candida albicans* from our analysis. EBV was also excluded due to the high prevalence in the adult human population. The detailed distribution of pathogens is shown in Table 2.

According to the detection of pathogens, we divided the patients into a single pathogen group (i.e., only influenza A virus) and a multipathogen group and compared the cytokine levels between the two groups, and no significant difference was found ((available here) Supplementary Table 5). Patients positive for other respiratory viruses had elevated IFN-*γ* levels (63.97 pg/ml and 44.48 pg/ml, *P* = 0.041), IL-10 levels (9.74 pg/ml and 3.89 pg/ml, *P* = 0.049), and decreased IL-22 levels (8.22 pg/ml and 14.02 pg/ml, *P* = 0.026) compared with patients negative for other respiratory viruses ((available here) Supplementary Table 6). However, no significant difference was found between patients positive and negative for bacteria or fungi ((available here) Supplementary Table 7).

### 3.3. Cytokine Profiles in Patients with Influenza A Pneumonia

**Figure 2:**
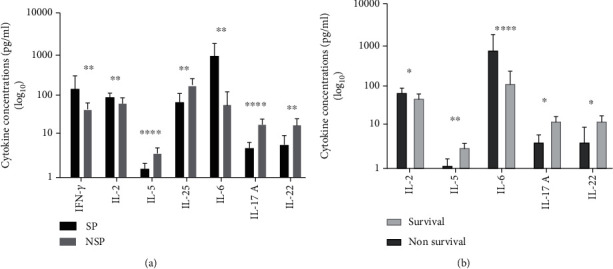
Levels of cytokines in different subgroups of patients with influenza A pneumonia. (a) Comparison of the levels of IFN-*γ*, IL-2, IL-5, IL-25, IL-6, IL-17A, and IL-22 in the NSP group (*n* = 25) and SP group (*n* = 10). (b) Comparison of the levels of IL-2, IL-5, IL-6, IL-17A, and IL-22 in the survival group (*n* = 30) and nonsurvival group (*n* = 5). ^∗^*P* < 0.05, ^∗∗^*P* < 0.01, ^∗∗∗∗^*P* < 0.0001. Abbreviations: SP: severe pneumonia; NSP: nonsevere pneumonia; IFN: interferon; IL: interleukin.

**Figure 3:**
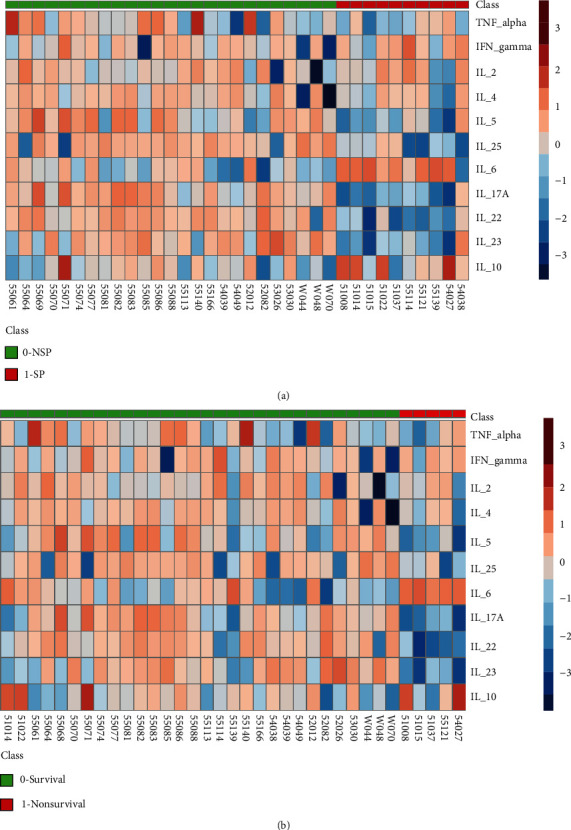
Overview of cytokine profiles in patients with influenza A pneumonia with different prognoses. (a) Patients were divided into the NSP group (*n* = 25, green) and SP group (*n* = 10, red). (b) Patients were divided into a survival group (*n* = 30, green) and a nonsurvival group (*n* = 5, red). Abbreviations: SP: severe pneumonia; NSP: nonsevere pneumonia; TNF: tumor necrosis factor; IL: interleukin; IFN: interferon.

We further analyzed the levels of 11 cytokines in different subgroups. As shown in Figure 2(a), the levels of IFN-*γ*, IL-2, and IL-6 were significantly higher in the NSP group and IL-5, IL-25, IL-17A, and IL-22 were significantly lower in the SP group (all *P* < 0.05). As shown in Figure 2(b), the levels of IL-2 and IL-6 were significantly higher in the nonsurvival group than in the survival group and IL-5, IL-17A, and IL-22 were significantly lower (all *P* < 0.05). The levels of IL-6 showed the most obvious difference, with approximately 40.5-fold and 47.7-fold increases in the SP group and nonsurvival group, respectively. No significant difference was observed for Treg cytokine (IL-10) in different subgroups. The levels of cytokines in each group are shown in Table 3. In general, patients with poor diagnosis (severe pneumonia or death) had specific cytokine profiles, with high Th1 cytokines and low Th2 cytokines, high IL-6, and low other Th17 cytokines. The expression profile is clearly shown in heatmaps (Figure 3). In addition, we found that the detection rate of multiple pathogens, especially other respiratory viruses, was higher in the severe pneumonia group (as shown in (available here) Supplementary Table 8), and the cytokine profiles of the three groups were mostly consistent ((available here) Supplementary Table 5 and Supplementary Table 6), which suggested that multiple pathogen infection may be an important factor in severe pneumonia.

### 3.4. Value of Cytokines in Predicting Prognosis in Patients with Influenza A Pneumonia

**Figure 4:**
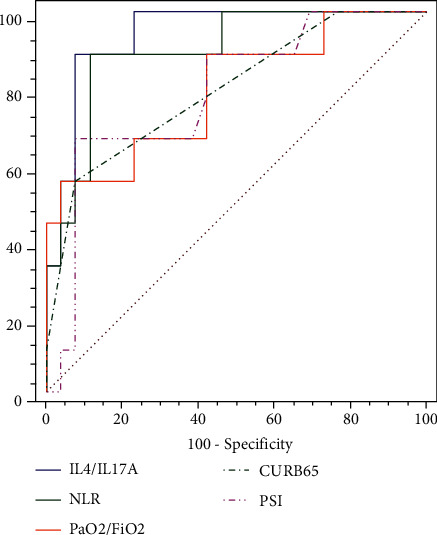
ROC curve analysis of various indicators to predict IPPV in patients with influenza A pneumonia. Abbreviations: NLR: neutrophil/lymphocyte ratio; IL: interleukin; PSI: pneumonia severity index.

To explore whether cytokines can be used as indicators of the severity of the disease, we further analyzed the levels of cytokines and the ratio of different cytokines and compared the ROC curves between them. The results showed that the IL-4/IL-17A ratio with a cutoff point > 4.97 pg/ml was a good predictor of IPPV risk in patients with influenza A pneumonia (AUC = 0.940, *P* < 0.001), with a sensitivity of 88.89% and specificity of 92.31%. The prediction capability of IL-4/IL-17A was greater than that of other commonly used indicators, including NLR (AUC = 0.897), oxygenation index (AUC = 0.795), CURB-65 (AUC = 0.795), and pneumonia severity index (PSI) (AUC = 0.786) (as shown in Figure 4 and Table 4). The value of IL-4/IL-17A in predicting severe influenza A pneumonia exhibited similar results, as shown in (available here) Supplementary Figure 1 and Supplementary Table 9.

To reveal the association between the IL-4/IL-17A ratio and the risk of IPPV, we further performed backward stepwise logistic regression analysis. A multivariate model adjusting for age, NLR, oxygenation index, CURB-65, and PSI suggested that the IL-4/IL-17A ratio (OR = 3.772, 95% CI, 1.188–11.975, *P* = 0.024) was an independent risk factor for IPPV. For each unit increase in the ratio, the risk of IPPV increased by 3.8 times (as shown in Table 5).

In addition to the IL-4/IL-17A ratio, four cytokine ratio and IL-6 levels were found to be good predictors of IPPV risk in patients with influenza A pneumonia (AUC = 0.987, 0.983, 0.944, 0.923, and 0.908, respectively, *P* < 0.001, as shown in (available here) Supplementary Table 10 and Supplementary Figure 2). They were also independent risk factors for IPPV, with OR (95% CI) of 1.217 (1.007–1.472), 1.482 (1.050–2.092), 1.702 (1.102–2.627), 1.004 (1.001–1.008), and 1.156 (1.020–1.311), respectively (*P* < 0.05), less than IL-4/IL-17A.

## 4. Discussion

In this study, we described the Th cell cytokine expression profiles, which comprised Th1, Th2, Th17, and Treg, cells, in the sputum supernatant samples of 35 patients with influenza A pneumonia. We found that high levels of IL-6, IFN-*γ*, and IL-2 and low levels of IL-5, IL-25, IL-17A, and IL-22 were found in the severe pneumonia group compared to the nonsevere pneumonia group. Similarly, high levels of IL-2 and IL-6 and low levels of IL-5, IL-17A, and IL-22 were observed in the nonsurvival group compared to the survival group. In brief, the expression pattern of high Th1, low Th2, high IL-6, and low other Th17 cytokines was associated with severe clinical conditions. In addition, a high IL-4/IL-17A ratio could be a good predictor for the necessity of IPPV in influenza A cases.

There have been previous studies focusing on cytokines in influenza. Overall, the majority cytokines were elevated in patients with influenza pneumonia compared with healthy individuals in peripheral and lung samples [22–24]. Further, increases in IL-6, IL-8, IL-10, IL-15, TNF-*α*, and IFN-*γ* have been reported in severe pneumonia compared with mild pneumonia [22, 25]. IFN-*γ*, TNF-*α*, IL-4, and IL-2 were associated with increased neutrophils [26]. Il-6 and IL-8 have been found to be associated with PaO2 in critical patients [27]. IL-13 was associated with intensive care unit (ICU) admission and protection against pneumonia. Meanwhile, the IFN-*γ*/IL-13 ratio may be used to predict the risk of pneumonia and ICU hospitalization [28]. These findings revealed the T-helper 1 and T-helper 17 responses in severe pneumonia, suggesting the potential of cytokines as biomarkers of disease severity.

It is generally recognized that during infections, Th1 cells mediate the immune response to resist invading pathogens, and many autoimmune responses are due to abnormal activation of Th1 cells. Th2 cells regulate immune responses against a variety of extracellular parasites and play an important role in asthma and other sensitization diseases [29, 30]. Th17 cells mainly regulate the immune response to extracellular pathogens, including bacteria and fungi, and induce organ-related autoimmune diseases [31].

IFN-*γ*, IL-2, and LT*α* are major cytokines produced by Th1 cells. IFN-*γ*, as a signature cytokine for CD4^+^ T cells, plays an essential role in activating macrophages to enhance their microbicidal activity [30]. It is released by cytotoxic lymphocytes and activated T cells to limit viral replication [32–34]. High levels of IFN-*γ* are related to the range of the decrease in viral titers [35]. A study using a mouse model showed that IFN was elevated transiently in the early stages of influenza infection [36]. IL-2 is crucial for the multiplication of T cells and memory formation of CD4^+^ and CD8^+^ cells. Previous studies showed that mice with an IL-2 knockout developed autoimmune symptoms [37], and that IL-2 blockage led to a rapid autoimmunity [38]. It has been suggested that IL-2 acts to control immune responses and sustain self-tolerance [39]. However, research has shown that IL-2 transfusion can induce prompt and intense pulmonary inflammation through natural killer cells [40], which is in accord with our findings that IL-2 levels were higher in the severe pneumonia group.

IL-6 is a versatile cytokine that activates B cells and accelerates the differentiation of Th0 cells into Th17 cells and regulatory T cells and is therefore an essential factor in the inflammatory response. Excessively high levels of IL-6 have been detected in several diseases, most commonly in rheumatoid arthritis (RA) and Castleman's disease, as well as in a variety of tumors [41]. In addition, over synthesis and persistent elevation of IL-6 can lead to a cytokine storm, which is thought to be an important manifestation of severe viral pneumonia such as influenza A pneumonia and COVID-19 [42, 43]. The immunomodulatory function is mediated by a complex of IL-6 and IL-6 receptors with gp130 [41, 44]. Thus, the current therapeutic agent, tocilizumab, mainly targets the IL-6 receptor and was originally used to treat chronic inflammatory diseases such as rheumatoid arthritis [45]. Recently, preliminary results from several studies showed that tocilizumab improved clinical outcomes and reduced mortality in patients with COVID-19 [43, 46].

IL-4, IL-5, and IL-25 are major cytokines produced by Th2 cells. Secretion of IL-4 cytokines by Th2 cells promotes Th2 cell differentiation, thus forming positive feedback on Th2 cell differentiation. IL-5 is primarily responsible for recruiting eosinophils. In addition, when the release of IL-5 is inhibited, the body's susceptibility to influenza A increases. Moreover, IL-5 can also inhibit neutrophil function. Therefore, when the expression of IL-5 is decreased, release of a large amount of myeloperoxidase by neutrophils can occur, aggravating lung injury [47].

The initial stage of influenza virus infection activates alveolar macrophages and monocytes, leading to an inflammatory cytokine response, mainly TNF-*α* and IL-6. IL-6 can promote the transformation of naive T cells into Th17 cells, releasing IL-17A and IL-22 [48]. IL-17A is associated with acute lung inflammation and injury [49, 50]. IL-22 is produced by natural killer cells and T cells and can promote the regeneration of epithelial cells. During severe influenza, IL-22 immunotherapy can enhance the integrity of lung tissue and reduce the systemic invasion of secondary bacteria [51, 52]. These two cytokines, especially IL-22, play a critical role in pathogen clearance. In our study, in the severe and death groups, there was a trend of high IL-6, low IL-17A, and IL-22, suggesting that downregulations of IL-22 and IL-17A may be critical in disease progression. The early immune response phase of influenza A was characterized by rapidly generalized lymphocytopenia, preferential loss of Th17 population, and T cell activation [53]. The mechanism may be that IL-21R signaling essentially inhibited the accumulation of IL-17^+^*γδ* T cells in the respiratory tract [54]. In addition, CCR2 restricted the induction of IL-17 by dendritic cells after influenza A infection [55]. In our study, IL-6 levels were elevated with decreased lymphocytes in the SP group. Thus, although IL-6 can promote the differentiation of Th17 cells and the secretion of IL-17A and IL-22, low levels of IL-17A and IL-22 are more likely due to lymphopenia.

In general, Th1 cells are necessary for the body to combat pathogens, but excessively elevated Th1 cytokines often indicate a heavier inflammatory response, which is related to cytokine storms. Th2 cells and Th17 cells also mediate the host's immune response to various pathogens, and low levels of Th2 and Th17 cytokines indicate a decline in the body's ability to eliminate pathogens. The combination of the two conditions indicates the poor prognosis of patients.

More importantly, this study provides theoretical support for the IL-4/IL-17A ratio to predict IPPV risk. IL-4 is not only a prototypical growth factor for B cells but also a key regulator of macrophage function [56]. Several studies have shown that IL-4-activated macrophages play an essential role in tissue repair and regeneration after injury [57, 58]. However, it is suggested that macrophages stimulated by IL-4 are more susceptible to the Ebola model virus [59]. Furthermore, a study has shown that when IL-4 levels are high, viral clearance in mice infected with influenza virus is delayed [60].

There are still many deficiencies in this study. First, the number of cases is not very large, resulting in our calculation results including regression models that may not being robust enough. Although the sample size was small, this was counterbalanced by the small *P* values related to many comparisons. However, it is possible that some other significant associations were missed due to the small sample size (type II error). Therefore, it is just a pilot study and systematic verification in larger populations, and the construction of animal models is needed. Similarly, a larger sample is especially required to assess the cytokine profile among patients with pure influenza A pneumonia infection compared with coinfected with other viruses and bacteria. A larger sample size will also make it possible to compare patient with influenza A pneumonia according to the presence or absence of co-morbidities. Second, we did not conduct longitudinal studies, and further dynamic observation of the expression of the IL4/IL-17A ratio with changes in the disease condition may be more conducive to the indepth understanding of the occurrence and development of the disease. Third, there was no control group, such as patients with chronic obstructive pulmonary disease (COPD) or bacterial pneumonia.

## 5. Conclusions

In summary, our study detected the cytokine profiles in the sputum of patients with influenza A pneumonia. We identified specific cytokine profiles in patients with severe pneumonia, which presented as high Th1 cytokines, low Th2 cytokines, high IL-6, and low other Th17 cytokines. The IL-4/IL-17A ratio has the potential to be a good predictor and an independent risk factor for IPPV.

## Figures and Tables

**Table 1:** Clinical characteristics of the patients.

	NSP (*n* = 25)	SP (*n* = 10)	*P*
Age, yrs	65.5 ± 19.2	60.5 ± 12.3	0.167
Male	15 (60%)	8 (80%)	0.434
BMI, kg/m^2^	21.4 ± 4.5	19.0 ± 4.3	0.166
Smoking history	6 (24.0%)	5 (50.0%)	0.227
Comorbidities	14 (56.0%)	1 (10.0%)	0.022
COPD	4 (16.0%)	0 (0.0%)	0.303
Bronchiectasis	4 (16.0%)	0 (0.0%)	0.303
Type 2 diabetes	7 (29.2%)	0 (0.0%)	0.084
Cardiovascular disease	4 (16.0%)	1 (10.0%)	1.000
Laboratory tests			
WBC, ×10^9^/L	5.60 (4.09-7.11)	4.41 (3.70-9.68)	0.733
NE, ×10^9^/L	3.57 (2.35-5.20)	3.91 (3.22-8.36)	0.240
LY, ×10^9^/L	1.37 ± 0.58	0.57 ± 0.54	0.001
NLR	2.71 (1.70-5.36)	9.86 (6.11-19.44)	<0.001
CRP, mg/L	28.70 (6.00-67.22)	96.31 (35.17-158.65)	0.009
PCT, *μ*g/L	0.15 (0.03-2.53)	1.25 (0.35-1.85)	0.339
PaO_2_/FiO_2_, mmHg	172.12 (136.6-342.79)	120.85 (82.88-169.09)	0.017
Outcome			
NIPPV	1 (4.0%)	7 (70.0%)	<0.001
IPPV	0 (0.0%)	9 (90.0%)	<0.001
ICU admission	0 (0.0%)	9 (90.0%)	<0.001
Total mortality	0 (0.0%)	5 (50.0%)	<0.001

Abbreviations: NSP: nonsevere pneumonia; SP: severe pneumonia; BMI: body mass index; COPD: chronic obstructive pulmonary disease; WBC: white blood cells; NE: neutrophils; LY: lymphocytes; NLR: neutrophil/lymphocyte ratio; CRP: c-reactive protein; PCT: procalcitonin; NIPPV: noninvasive positive pressure ventilation; IPPV: intermittent positive pressure ventilation; ICU: intensive care unit.

**Table 2:** Distribution of pathogens detected in patients with influenza A pneumonia.

Pathogens		Cases, %
Bacteria	*Streptococcus pneumoniae*	2 (5.7%)
	*Pseudomonas aeruginosa*	2 (5.7%)
	*Staphylococcus haemolyticus*	2 (5.7%)
	*Staphylococcus aureus*	1 (2.9%)
	*Staphylococcus epidermidis*	1 (2.9%)
	*Staphylococcus hominis*	1 (2.9%)
	*Acinetobacter baumannii*	1 (2.9%)
	*Haemophilus influenzae*	1 (2.9%)
	*Stenotrophomonas maltophilia*	1 (2.9%)
Virus	Epstein-Barr virus (EBV)^a^	13 (37.1%)
	Adenovirus	6 (17.1%)
	Influenza B virus	2 (5.7%)
	Herpes simplex virus- (HSV-) 1	2 (5.7%)
	Cytomegalovirus	2 (5.7%)
	Varicella zoster virus (VZV)	1 (2.9%)
	Human papilloma virus- (HPV-) 16	1 (2.9%)
	Rhinovirus	1 (2.9%)
Fungus	*Candida albicans* ^a^	14 (40.0%)
	*Aspergillus genus*	5 (14.3%)
	*Candida tropicalis*	1 (2.9%)
	*Candida krusei*	1 (2.9%)

^a^EBV and *Candida albicans* were excluded from our analysis due to the high prevalence in the adult human population.

**Table 3:** Levels of 11 cytokines in different subgroups of patients with influenza A pneumonia.

	NSP (*n* = 25)	SP (*n* = 10)	*P*	Survival (*n* = 30)	Nonsurvival (*n* = 5)	*P*
TNF-*α*, pg/ml	18.51 (6.62-33.31)	10.03 (5.71-64.36)	0.627	17.64 (5.81-38.13)	13.60 (6.20-178.24)	0.909
IFN-*γ*, pg/ml	41.97 (22.56-56.69)	69.36 (53.27-153.57)	0.009	48.92 (25.89-64.64)	98.74 (32.91-557.81)	0.185
IL-2, pg/ml	60.03 (37.57-88.18)	103.27 (77.85-119.44)	0.008	67.26 (45.19-97.71)	105.47 (88.41-128.77)	0.043
IL-4, pg/ml	31.73 (21.10-42.48)	36.61 (28.65-43.02)	0.483	32.95 (21.42-40.03)	36.64 (30.19-49.81)	0.321
IL-5, pg/ml	2.47 (1.70-6.67)	1.52 (1.26-1.70)	< 0.0001	2.03 (1.59-4.83)	1.44 (0.71-1.59)	0.001
IL-25, pg/ml	133.39 (103.49-261.53)	83.87 (0.71-120.55)	0.009	133.39 (93.81-229.56)	83.87 (42.29-146.21)	0.202
IL-6, pg/ml	14.05 (6.79-65.12)	569.14 (80.47-1826.25)	0.002	18.13 (6.91-99.67)	865.65 (272.08-2979.5)	< 0.0001
IL-17A, pg/ml	14.87 (7.27-33.95)	4.79 (3.67-8.19)	< 0.0001	11.32 (5.93-32.55)	4.56 (3.47-6.96)	0.012
IL-22, pg/ml	12.62 (8.98-32.82)	7.08 (0.71-10.72)	0.006	11.50 (8.20-26.96)	0.71 (0.71-11.59)	0.043
IL-23, pg/ml	194.89 (129.76-340.71)	110.42 (77.91-198.94)	0.059	183.23 (112.94-302.15)	105.64 (63.20-186.44)	0.086
IL-10, pg/ml	4.24 (0.71-10.21)	22.33 (3.74-88.01)	0.065	4.60 (0.71-11.06)	8.11 (3.37-526.30)	0.277

Abbreviations: NSP: nonsevere pneumonia; SP: severe pneumonia; TNF: tumor necrosis factor; IFN: interferon; IL: interleukin.

**Table 4:** ROC curve analysis of various indicators to predict IPPV in patients with influenza A pneumonia.

	Cutoff points	AUC	95% CI	*P*	Sensitivity (%)	Specificity (%)
IL-4/IL-17A	>4.97	0.940	0.805-0.992	<0.001	88.89	92.31
NLR	>6.57	0.897	0.748-0.974	<0.001	88.89	88.46
PaO_2_/FiO_2_	≤110	0.795	0.625-0.912	0.002	55.56	96.15
CURB-65	>1	0.795	0.625-0.912	<0.001	55.56	92.31
PSI	>102	0.786	0.615-0.906	0.002	66.67	92.31

Abbreviations: IPPV: intermittent positive pressure ventilation; AUC: area under curve; CI: confidence interval; IL: interleukin; NLR: neutrophil/lymphocyte ratio; PSI: pneumonia severity index.

**Table 5:** Univariate and multivariate analysis of the relationship between various parameters and IPPV.

	Univariate analysis		Multivariate logistic regression	
	OR (95% CI)	*P*	OR (95% CI)	*P*
Age	0.984 (0.943-1.028)	0.469		
NLR	1.385 (1.086–1.786)	0.009		
PaO_2_/FiO_2_	0.985 (0.970–1.000)	0.052		
IL-4/IL-17A	2.143 (1.289–3.564)	0.003	3.772 (1.188–11.975)	0.024
CURB-65	11.211 (1.845–68.105)	0.009		
PSI	2.227 (0.933–5.315)	0.071		

Abbreviations: IPPV: intermittent positive pressure ventilation; CI: confidence interval; NLR: neutrophil/lymphocyte ratio; IL: interleukin; PSI: pneumonia severity index.

## Data Availability

The datasets used during the current study, including clinical data and cytokine levels of participants are available from the corresponding author on reasonable request, and we will make a reply as soon as possible.

## Supplementary Materials

Supplementary Materials Figure 1: ROC curve analysis of various indicators to predict severe influenza A pneumonia. Figure 2: ROC curve analysis of various indicators to predict IPPV in patients with influenza A pneumonia. Table 1: median levels and percentages of measurements below the lower LOD for 12 cytokines. Table 2: index of each reaction tank for 5 chips. Table 3: clinical characteristics of patients positive for 2009H1N1 or H3N1. Table 4: levels of 11 cytokines of patients positive for 2009H1N1 or H3N1. Table 5: levels of 11 cytokines in multipathogen-positive group and single pathogen-positive group. Table 6: levels of 11 cytokines in other respiratory virus-positive groups and other respiratory virus-negative groups. Table 7: levels of 11 cytokines in different subgroups of patients. Table 8: distribution of pathogens in the SP group and NSP group. Table 9: ROC curve analysis of various indicators to predict severe influenza A pneumonia. Table 10: ROC curve analysis of various indicators to predict IPPV in patients with influenza A pneumonia.

## References

[1] Xu X. W., Wu X. X., Jiang X. G. (2020). Clinical findings in a group of patients infected with the 2019 novel coronavirus (SARS-Cov-2) outside of Wuhan, China: retrospective case series. *BMJ*.

[2] Yang X., Yu Y., Xu J. (2020). Clinical course and outcomes of critically ill patients with SARS-CoV-2 pneumonia in Wuhan, China: a single-centered, retrospective, observational study. *The Lancet Respiratory Medicine*.

[3] Thompson W. W., Weintraub E., Dhankhar P. (2009). Estimates of US influenza-associated deaths made using four different methods. *Influenza and Other Respiratory Viruses*.

[4] Darwish I., Mubareka S., Liles W. C. (2011). Immunomodulatory therapy for severe influenza. *Expert Review of Anti-Infective Therapy*.

[5] To K. K., Hung I. F., Li I. W. (2010). Delayed clearance of viral load and marked cytokine activation in severe cases of pandemic H1N1 2009 influenza virus infection. *Clinical Infectious Diseases*.

[6] Beigel J. H., Farrar J., Han A. M., Hayden F. G., Hyer R., de Jong M. D. (2005). Avian influenza a (H5N1) infection in humans. *The New England Journal of Medicine*.

[7] Liu Q., Zhou Y. H., Yang Z. Q. (2016). The cytokine storm of severe influenza and development of immunomodulatory therapy. *Cellular & Molecular Immunology*.

[8] Oldstone M. B., Rosen H. (2014). Cytokine storm plays a direct role in the morbidity and mortality from influenza virus infection and is chemically treatable with a single sphingosine-1-phosphate agonist molecule. *Current Topics in Microbiology and Immunology*.

[9] Tisoncik J. R., Korth M. J., Simmons C. P., Farrar J., Martin T. R., Katze M. G. (2012). Into the eye of the cytokine storm. *Microbiology and Molecular Biology Reviews*.

[10] Teijaro J. R. (2014). The role of cytokine responses during influenza virus pathogenesis and potential therapeutic options. *Current Topics in Microbiology and Immunology*.

[11] Uchide N., Toyoda H. (2011). Antioxidant therapy as a potential approach to severe influenza-associated complications. *Molecules*.

[12] Hung I. F. N., To K. K. W., Lee C. K. (2013). Hyperimmune IV immunoglobulin treatment: a multicenter double-blind randomized controlled trial for patients with severe 2009 influenza A(H1N1) infection. *Chest*.

[13] Hui D. S., Lee N., Chan P. K., Beigel J. H. (2018). The role of adjuvant immunomodulatory agents for treatment of severe influenza. *Antiviral Research*.

[14] Cao B., Gao H., Zhou B. (2016). Adjuvant corticosteroid treatment in adults with influenza A (H7N9) viral pneumonia. *Critical Care Medicine*.

[15] on behalf of the GETGAG Study Group, Moreno G., Rodríguez A. (2018). Corticosteroid treatment in critically ill patients with severe influenza pneumonia: a propensity score matching study. *Intensive Care Medicine*.

[16] Niederman M. S., Mandell L. A., Anzueto A. (2001). Guidelines for the management of adults with community-acquired pneumonia. Diagnosis, assessment of severity, antimicrobial therapy, and prevention. *American Journal of Respiratory and Critical Care Medicine*.

[17] Pavord I. D., Pizzichini M. M., Pizzichini E., Hargreave F. E. (1997). The use of induced sputum to investigate airway inflammation. *Thorax*.

[18] Kelly M. M., Keatings V., Leigh R. (2002). Analysis of fluid-phase mediators. *The European Respiratory Journal. Supplement*.

[19] Yu Y., Zhao L., Xie Y. (2020). Th1/Th17 cytokine profiles are associated with disease severity and exacerbation frequency in COPD patients. *International Journal of Chronic Obstructive Pulmonary Disease*.

[20] Lubin J. H., Colt J. S., Camann D. (2004). Epidemiologic evaluation of measurement data in the presence of detection limits. *Environmental Health Perspectives*.

[21] Hornung R. W., Reed L. D. (1990). Estimation of average concentration in the presence of nondetectable values. *Applied Occupational and Environmental Hygiene*.

[22] Bradley-Stewart A., Jolly L., Adamson W. (2013). Cytokine responses in patients with mild or severe influenza a(H1N1)pdm09. *Journal of Clinical Virology*.

[23] Martinez-Ocaña J., Olivo-Diaz A., Salazar-Dominguez T. (2013). Plasma cytokine levels and cytokine gene polymorphisms in Mexican patients during the influenza pandemic a(H1N1)pdm09. *Journal of Clinical Virology*.

[24] Zúñiga J., Torres M., Romo J. (2011). Inflammatory profiles in severe pneumonia associated with the pandemic influenza A/H1N1 virus isolated in Mexico City. *Autoimmunity*.

[25] Hagau N., Slavcovici A., Gonganau D. N. (2010). Clinical aspects and cytokine response in severe H1N1 influenza A virus infection. *Critical Care*.

[26] Matsumoto Y., Kawamura Y., Nakai H. (2012). Cytokine and chemokine responses in pediatric patients with severe pneumonia associated with pandemic A/H1N1/2009 influenza virus. *Microbiology and Immunology*.

[27] Bermejo-Martin J. F., de Lejarazu R. O., Pumarola T. (2009). Th1 and Th17 hypercytokinemia as early host response signature in severe pandemic influenza. *Critical Care*.

[28] L’Huillier A. G., Ferreira V. H., Hirzel C. (2019). Cytokine profiles and severity of influenza infection in transplant recipients. *The Journal of Infectious Diseases*.

[29] Mosmann T. R., Coffman R. L. (1989). TH1 and TH2 cells: different patterns of lymphokine secretion lead to different functional properties. *Annual Review of Immunology*.

[30] Paul W. E., Seder R. A. (1994). Lymphocyte responses and cytokines. *Cell*.

[31] Weaver C. T., Harrington L. E., Mangan P. R., Gavrieli M., Murphy K. M. (2006). Th17: an effector CD4 T cell lineage with regulatory T cell ties. *Immunity*.

[32] Yamada Y. K., Meager A., Yamada A., Ennis F. A. (1986). Human interferon alpha and gamma production by lymphocytes during the generation of influenza virus-specific cytotoxic T lymphocytes. *The Journal of General Virology*.

[33] Mbawuike I. N., Fujihashi K., DiFabio S. (1999). Human interleukin-12 enhances interferon-gamma-producing influenza-specific memory CD8+ cytotoxic T lymphocytes. *The Journal of Infectious Diseases*.

[34] McMichael A. J., Gotch F. M., Noble G. R., Beare P. A. (1983). Cytotoxic T-cell immunity to influenza. *The New England Journal of Medicine*.

[35] Kaiser L., Fritz R. S., Straus S. E., Gubareva L., Hayden F. G. (2001). Symptom pathogenesis during acute influenza: interleukin-6 and other cytokine responses. *Journal of Medical Virology*.

[36] Lv J., Wang D., Hua Y. H. (2014). Pulmonary immune responses to 2009 pandemic influenza A (H1N1) virus in mice. *BMC Infectious Diseases*.

[37] Suzuki H., Kundig T. M., Furlonger C. (1995). Deregulated T cell activation and autoimmunity in mice lacking interleukin-2 receptor beta. *Science*.

[38] Setoguchi R., Hori S., Takahashi T., Sakaguchi S. (2005). Homeostatic maintenance of natural Foxp3(+) CD25(+) CD4(+) regulatory T cells by interleukin (IL)-2 and induction of autoimmune disease by IL-2 neutralization. *The Journal of Experimental Medicine*.

[39] Abbas A. K., Trotta E., R Simeonov D., Marson A., Bluestone J. A. (2018). Revisiting IL-2: Biology and therapeutic prospects. *Sci Immunol*.

[40] McKinstry K. K., Alam F., Flores-Malavet V. (2019). Memory CD4 T cell-derived IL-2 synergizes with viral infection to exacerbate lung inflammation. *PLoS Pathogens*.

[41] Kang S., Narazaki M., Metwally H., Kishimoto T. (2020). Historical overview of the interleukin-6 family cytokine. *The Journal of Experimental Medicine*.

[42] Fajgenbaum D. C., June C. H. (2020). Cytokine Storm. *The New England Journal of Medicine*.

[43] Zhang S., Li L., Shen A., Chen Y., Qi Z. (2020). Rational use of tocilizumab in the treatment of novel coronavirus pneumonia. *Clinical Drug Investigation*.

[44] Taniguchi K., Karin M. (2014). IL-6 and related cytokines as the critical lynchpins between inflammation and cancer. *Seminars in Immunology*.

[45] Tanaka T., Narazaki M., Kishimoto T. (2018). Interleukin (IL-6) immunotherapy. *Cold Spring Harbor Perspectives in Biology*.

[46] Xu X., Han M., Li T. (2020). Effective treatment of severe COVID-19 patients with tocilizumab. *Proceedings of the National Academy of Sciences of the United States of America*.

[47] Califano D., Furuya Y., Roberts S., Avram D., McKenzie A. N. J., Metzger D. W. (2018). IFN-gamma increases susceptibility to influenza A infection through suppression of group II innate lymphoid cells. *Mucosal Immunology*.

[48] Zhu J., Paul W. E. (2008). CD4 T cells: fates, functions, and faults. *Blood*.

[49] Li C., Yang P., Sun Y. (2012). IL-17 response mediates acute lung injury induced by the 2009 pandemic influenza A (H1N1) virus. *Cell Research*.

[50] Chen T., Qiu H., Zhao M. M. (2019). IL-17A contributes to HSV1 infection-induced acute lung injury in a mouse model of pulmonary fibrosis. *Journal of Cellular and Molecular Medicine*.

[51] Ivanov S., Renneson J., Fontaine J. (2013). Interleukin-22 reduces lung inflammation during influenza A virus infection and protects against secondary bacterial infection. *Journal of Virology*.

[52] Barthelemy A., Sencio V., Soulard D. (2018). Interleukin-22 immunotherapy during severe influenza enhances lung tissue integrity and reduces secondary bacterial systemic invasion. *Infection and Immunity*.

[53] Jiang T. J., Zhang J. Y., Li W. G. (2010). Preferential loss of Th17 cells is associated with CD4 T cell activation in patients with 2009 pandemic H1N1 swine-origin influenza A infection. *Clinical Immunology*.

[54] Moser E. K., Sun J., Kim T. S., Braciale T. J. (2015). IL-21R signaling suppresses IL-17+ gamma delta T cell responses and production of IL-17 related cytokines in the lung at steady state and after Influenza A virus infection. *PLoS One*.

[55] Gurczynski S. J., Nathani N., Warheit-Niemi H. I. (2019). CCR2 mediates increased susceptibility to post-H1N1 bacterial pneumonia by limiting dendritic cell induction of IL-17. *Mucosal Immunology*.

[56] Stein M., Keshav S., Harris N., Gordon S. (1992). Interleukin 4 potently enhances murine macrophage mannose receptor activity: a marker of alternative immunologic macrophage activation. *The Journal of Experimental Medicine*.

[57] Minutti C. M., Knipper J. A., Allen J. E., Zaiss D. M. (2017). Tissue-specific contribution of macrophages to wound healing. *Seminars in Cell & Developmental Biology*.

[58] Van Dyken S. J., Locksley R. M. (2013). Interleukin-4- and interleukin-13-mediated alternatively activated macrophages: roles in homeostasis and disease. *Annual Review of Immunology*.

[59] Rogers K. J., Brunton B., Mallinger L. (2019). IL-4/IL-13 polarization of macrophages enhances Ebola virus glycoprotein-dependent infection. *PLoS Neglected Tropical Diseases*.

[60] Bot A., Holz A., Christen U. (2000). Local IL-4 expression in the lung reduces pulmonary influenza-virus-specific secondary cytotoxic T cell responses. *Virology*.

