# Supplementary material for: Specific Cytokine Profiles Predict the Severity of Influenza A Pneumonia: A Prospectively Multicenter Pilot Study
Source: Biomed Res Int. 2021 Oct 13;2021:9533044. doi: 10.1155/2021/9533044 (PMC8528594; doi:10.1155/2021/9533044)
Supplement: Supplementary Materials — Figure 1: ROC curve analysis of various indicators to predict severe influenza A pneumonia. Figure 2: ROC curve analysis of various indicators to predict IPPV in patients with influenza A pneumonia. Table 1: median levels and percentages of measurements below the lower LOD for 12 cytokines. Table 2: index of each reaction tank for 5 chips. Table 3: clinical characteristics of patients positive for 2009H1N1 or H3N1. Table 4: levels of 11 cytokines of patients positive for 2009H1N1 or H3N1. Table 5: levels of 11 cytokines in multipathogen-positive group and single pathogen-positive group. Table 6: levels of 11 cytokines in other respiratory virus-positive groups and other respiratory virus-negative groups. Table 7: levels of 11 cytokines in different subgroups of patients. Table 8: distribution of pathogens in the SP group and NSP group. Table 9: ROC curve analysis of various indicators to predict severe influenza A pneumonia. Table 10: ROC curve analysis of various indicators to predict IPPV in patients with influenza A pneumonia. [file 9533044.f1.docx]

***Supplementary Material***

**Diagnosis of CAP and severe pneumonia**

CAP was defined by the following criteria: (1) a chest radiograph showing either new patchy infiltrates, leaf or segment consolidation, ground-glass opacity, or interstitial change; (2) at least one of the following signs: (a) the presence of cough, sputum production, and dyspnea; (b) core body temperature > 38.0°C; (c) auscultatory findings of abnormal breath sounds and rales; (d) peripheral white blood cell counts > 10 × 10^9^/L or < 4 × 10^9^/L; and (3) symptom onset that began in the community rather than in a healthcare setting(1).

Severe pneumonia was diagnosed by the presence of at least one major criterion or at least three minor criteria. Major criteria consist of the presence of acute respiratory failure requiring invasive mechanical ventilation and septic shock with the need for vasopressors. Minor criteria consisted of respiratory rate ≥ 30 breaths per min, PaO_2_/FiO_2_ ratio ≤ 250, multilobar infiltrates, confusion/disorientation, uremia (blood urea nitrogen level ≥ 20 mg/dL), and systolic blood pressure < 90 mmHg.(2, 3)

**Exclusion criteria**

The exclusion criteria were age < 18 years or the presence of any of the following: pregnancy, other significant respiratory diseases (e.g., active pulmonary tuberculosis, interstitial lung disease, or lung cancer), immunosuppressive condition, or chronic inflammatory disease (e.g., inflammatory bowel disease or connective tissue diseases).

**Extraction and purification of microbial DNA**

Sputum sediments suspended in 300 μL of lysis buffer were added to EP tubes containing zirconium beads. After vibrating at 1500 rpm and 95℃ for 10 minutes, the samples were centrifuged at 12000 rpm for 1 minute. The supernatants (200-250 μL) were transferred to new EP tubes and then fully mixed with 10 μL of Proteinase K. The mixtures were heated at 70℃ for 10 minutes and then fully mixed with 750 μL of binding buffer and 15 μL of magnetic beads. The samples were placed on a magnetic stand and washed with 500 μL of Wash Buffer I, 500 μL of Wash Buffer II, 500 μL of Wash Buffer II, and 550 μL of Wash Buffer III, successively. Then, the magnetic beads were suspended in elution buffer and vibrated twice at 300 rpm and 55℃ for 10 minutes. The eluents were collected (approximately 200-250 μL) in new EP tubes. After determination of DNA concentration and purity, the samples were stored at -80℃ until analysis.

**Extraction of viral nucleic acid**

Two hundred microliters of sputum supernatant was added to EP tubes along with 25 μL of protease and 200 μL of a mixture of carrier RNA, buffer AL, and buffer AVE. After incubation at 56℃ for 15 minutes, 250 μL of ethanol (96–100%) was added to the samples. The mixtures were carefully applied onto QIAamp MinElute columns. The columns were centrifuged at 8000 rpm for 1 minute, and the collection tubes containing the filtrate were discarded. Similar to the above steps, 500 μL of buffer AW1, 500 μL of buffer AW2, and 500 μL of ethanol (96–100%) were successively applied to the columns and centrifuged. The columns were dried by centrifugation (14000 rpm for 3 minutes) and heating (56℃ for 3 minutes). AVE buffer (80 μL) was carefully applied to the center of the membrane. After incubation (room temperature for 1 minute) and centrifugation (14000 rpm for 1 minute), the filtrates were collected in EP tubes and stored at -80℃ until analysis.

**Detection of Respiratory Pathogens**

Nucleic acid samples from each patient were detected for 76 kinds of common respiratory pathogens (35 kinds of bacteria, 3 kinds of atypical pathogens, 20 kinds of fungi, and 18 kinds of viruses) by 5 chips using the isothermal amplification chip method. Twenty-four reaction tanks contained a specific set of primers in each chip (as shown in Supplementary Table 2). Reaction systems were prepared as instructed by the manufacturer and loaded in reaction tanks of each chip. Chips were centrifuged (for chips No. 1, 2, 3 and 4, 6000 rpm for the 30 s at room temperature; for chip No. 5, 6000 rpm for 90 s at 40℃) and then put into an isothermal amplification nucleic acid analyzer for microfluidic chips (RTisochipTM-A, CapitalBio Corporation, Beijing, China). Chips No. 1, 2, 3, and 4 reacted for 50 minutes at 65℃, while chip No. 5 reacted for 40 minutes at 41℃. The detection results were interpreted according to amplification curves. For chips No. 1, 2, 3, and 4, positive criteria included (1) time positive (Tp) ≤ 40 minutes and (2) the presence of a standard S-shaped curve. Positive criteria for chip No. 5 was the presence of Tp.

**Supplementary Figure 1.** ROC curve analysis of various indicators to predict severe influenza A pneumonia.

**Abbreviations:** NLR, neutrophil/lymphocyte ratio; IL, interleukin; CURB-65, confusion, urea, respiratory rate, blood pressure, and age ≥ 65 years old; PSI, pneumonia severity index.

**Supplementary Figure 2.** ROC curve analysis of various indicators to predict IPPV in patients with influenza A pneumonia.

**Abbreviations:** IL, interleukin; TNF, tumor necrosis factor; IFN, interferon.

**Supplementary Table 1.** Median levels and percentages of measurements below the lower LOD for 12 cytokines

|  | **< lower LOD, %** | **Median level, pg/ml** |
| --- | --- | --- |
| TNF-α | 2.9 | 50.84 |
| IFN-γ | 8.6 | 16.12 |
| IL-2 | 5.7 | 70.11 |
| IL-4 | 5.7 | 34.17 |
| IL-5 | 5.7 | 2.03 |
| IL-25 | 17.1 | 133.39 |
| IL-6 | 8.6 | 27.80 |
| IL-17A | 0 | 8.89 |
| IL-21 | 80 | - |
| IL-22 | 14.3 | 10.37 |
| IL-23 | 0 | 179.35 |
| IL-10 | 25.7 | 5.31 |

**Abbreviations:** LOD, limit of detection; TNF, tumor necrosis factor; IFN, interferon; IL, interleukin.

**Supplementary Table 2.** Index of each reaction tank for 5 chips

| **Chips** | **Reaction tank** | **Index** | **Reaction tank** | **Index** | **Reaction tank** | **Index** |
| --- | --- | --- | --- | --- | --- | --- |
| **No. 1** | 1 | PC | 9 | *Stenotrophomonas maltophilia* | 17 | - |
|  | 2 | IC | 10 | *Mycobacterium tuberculosis* | 18 | - |
|  | 3 | *Staphylococcus aureus* | 11 | *Chlamydia pneumoniae* | 19 | *Streptococcus pyogenes* |
|  | 4 | *Streptococcus pneumoniae* | 12 | *Mycoplasma pneumoniae* | 20 | *Proteus mirabilis* |
|  | 5 | *Klebsiella pneumoniae* | 13 | *Legionella pneumophila* | 21 | *Enterobacter aerogenes* |
|  | 6 | *Acinetobacter baumannii* | 14 | *Escherichia coli* | 22 | - |
|  | 7 | *Pseudomonas aeruginosa* | 15 | *Enterococcus faecalis* | 23 | NC |
|  | 8 | *Haemophilus influenzae* | 16 | *Enterococcus faecium* | 24 | BC |
| **No. 2** | 1 | PC | 9 | *Borrelia burgdorferi* | 17 | *Mycobacterium kansasii* |
|  | 2 | IC | 10 | *Leptospira* | 18 | *Mycobateria gordonae* |
|  | 3 | *Staphylococcus epidermidis* | 11 | *Streptococcus agalactiae* | 19 | *Mycobacterium scrofulaceum* |
|  | 4 | *Staphylococcus capitis* | 12 | *Streptococcus dysgalactiae* | 20 | *Mycodacterium chelonei-abscessus* |
|  | 5 | *Staphylococcus hominis* | 13 | *Brucella abortus* | 21 | *Mycobacterium marinum-ulcerans* |
|  | 6 | *Staphylococcus haemolyticus* | 14 | *Staphylococcus sciuri* | 22 | *Mycobacterium phlei* |
|  | 7 | *Neisseria meningitidis* | 15 | *Clostridium perfringens* | 23 | *Mycobacterium gilvum* |
|  | 8 | *Treponema pallidum* | 16 | *Mycobacterium avium-*intracellular | 24 | NC |
| **No. 3** | 1 | PC | 9 | *Aspergillus flavus* | 17 | *Rhizomucor pusillus* |
|  | 2 | IC | 10 | *Aspergillus niger* | 18 | *Aspergillus ustus* |
|  | 3 | *Candida albicans* | 11 | *Aspergillus terreus* | 19 | *Pneumocystis jirovecii* |
|  | 4 | *Candida tropicalis* | 12 | *Aspergillus nidulans* | 20 | *Mucor circinelloides* |
|  | 5 | *Candida parapsilosis* | 13 | *Cryptococcus neoformans* | 21 | *Histoplasma capsulatum* |
|  | 6 | *Candida krusei* | 14 | *Cryptococcus gattii* | 22 | *Penicillium marneffei* |
|  | 7 | *Candida glabrata* | 15 | *Rhizopus oryzae* | 23 | NC |
|  | 8 | *Aspergillus fumigatus* | 16 | *Lichtheimia corymbifera* | 24 | BC |
| **No. 4** | 1 | PC | 9 | Epstein-Barr virus | 17 | Simian virus |
|  | 2 | IC | 10 | Herpes simplex virus 1 | 18 | - |
|  | 3 | Adenovirus | 11 | Herpes simplex virus 2 | 19 | - |
|  | 4 | Adenovirus 2 | 12 | Cytomegalovirus | 20 | NC |
|  | 5 | Adenovirus 31 | 13 | Varicella-zoster virus | 21 | BC |
|  | 6 | Adenovirus 40 | 14 | Human herpesvirus 8 | 22 | BC |
|  | 7 | Adenovirus 41 | 15 | Human papillomavirus 16 | 23 | BC |
|  | 8 | Adenovirus 7 | 16 | Hepatitis B virus | 24 | BC |
| **No. 5** | 1 | NC | 9 | Respiratory syncytial virus | 17 | Parainfluenza virus III |
|  | 2 | PC | 10 | Rhinovirus | 18 | Parainfluenza virus IV |
|  | 3 | Influenza A virus | 11 | Enterovirus 71 | 19 | Human metapneumovirus |
|  | 4 | Influenza A virus 2009H1 | 12 | Coxsackie virus A16 | 20 | Coronavirus OC43/HKU1 |
|  | 5 | Influenza A virus seasonal H1 | 13 | Coxsackie virus A6 | 21 | Coronavirus NL63/229E |
|  | 6 | Influenza A virus seasonal H3 | 14 | Adenovirus B | 22 | IC |
|  | 7 | Influenza A virus H7 | 15 | Parainfluenza virus I | 23 | NC |
|  | 8 | Influenza B virus | 16 | Parainfluenza virus II | 24 | BC |

**Abbreviations:** PC, positive control; IC, internal control; NC, negative control; BC, blank control.

**Supplementary Table 3.** Clinical characteristics of patients positive for 2009H1N1 or H3N1

|  | **2009H1N1**  **(n = 10)** | **H3N1**  **(n = 15)** | ***P*** |
| --- | --- | --- | --- |
| Age, yrs | 59.70 ± 14.8 | 72.1 ± 15.7 | 0.060 |
| Male | 9 (90.0%) | 8 (53.3%) | 0.088 |
| BMI, kg/m^2^ | 19.5 ± 5.0 | 21.8 ± 3.9 | 0.211 |
| Smoking history | 6 (60.0%) | 3 (20.0%) | 0.087 |
| Comorbidities |  |  |  |
| COPD | 0 (0.0%) | 3 (20.0%) | 0.250 |
| Bronchiectasis | 1 (10.0%) | 1 (6.7%) | 1.000 |
| Type 2 diabetes | 0 (0.0%) | 5 (33.3%) | 0.061 |
| Cardiovascular disease | 1 (10.0%) | 2 (13.3%) | 1.000 |
| Laboratory tests |  |  |  |
| WBC, ×10^9^/L | 4.43 (3.46-6.41) | 5.40 (4.30-7.50) | 0.196 |
| NE, ×10^9^/L | 3.69 (1.84-4.95) | 3.80 (2.80-4.60) | 0.849 |
| LY, ×10^9^/L | 0.8 ± 0.5 | 1.4 ± 0.7 | 0.024 |
| NLR | 4.91 (2.16-12.54) | 3.98 (1.71-6.57) | 0.461 |
| CRP, mg/L | 67.22 (8.24-158.65) | 27.70 (6.21-90.50) | 0.238 |
| PCT, μg/L | 0.99 (0.02-1.99) | 0.11 (0.03-1.60) | 0.935 |
| PaO_2_/FiO_2_, mmHg | 204.58 (107.44-329.70) | 148.33 (116.75-246.19) | 0.683 |
| Outcome |  |  |  |
| NIPPV | 3 (30.0%) | 1 (6.7%) | 0.267 |
| IPPV | 4 (40.0%) | 2 (13.3%) | 0.175 |
| ICU admission | 4 (40.0%) | 2 (13.3%) | 0.175 |
| Total mortality | 3 (30.0%) | 1 (6.7%) | 0.267 |

**Abbreviations:** BMI, body mass index; COPD, chronic obstructive pulmonary disease; WBC, white blood cells; NE, neutrophils; LY, lymphocytes; NLR, neutrophil/lymphocyte ratio; CRP, c-reactive protein; PCT, procalcitonin; NIPPV, noninvasive positive pressure ventilation; IPPV, intermittent positive pressure ventilation; ICU, intensive care unit.

**Supplementary Table 4.** Levels of 11 cytokines of patients positive for 2009H1N1 or H3N1

|  | **2009H1N1**  **(n = 10)** | **H3N1**  **(n = 15)** | ***P*** |
| --- | --- | --- | --- |
| TNF-α, pg/ml | 6.94 (5.57-168.20) | 18.51 (13.31-31.44) | 0.461 |
| IFN-γ, pg/ml | 63.88 (37.72-153.57) | 50.84 (33.44-66.65) | 0.338 |
| IL-2, pg/ml | 95.30 (71.34-127.79) | 58.57 (34.49-75.76) | 0.019 |
| IL-4, pg/ml | 37.81 (28.65-47.90) | 36.58 (29.27-45.99) | 0.807 |
| IL-5, pg/ml | 1.59 (1.26-2.14) | 4.21 (2.03-7.06) | 0.004 |
| IL-25, pg/ml | 138.60 (102.40-198.86) | 133.39 (113.16-220.86) | 1.000 |
| IL-6, pg/ml | 101.32 (1.23-866.50) | 22.97 (9.76-105.35) | 0.892 |
| IL-17A, pg/ml | 7.27 (4.38-20.42) | 19.08 (7.95-32.27) | 0.160 |
| IL-22, pg/ml | 10.94 (9.00-17.51) | 11.50 (7.03-18.18) | 0.849 |
| IL-23, pg/ml | 148.93 (103.28-292.26) | 194.89 (163.91-293.25) | 0.807 |
| IL-10, pg/ml | 3.91 (0.71-63.55) | 6.03 (2.54-11.62) | 0.892 |

**Abbreviations:** TNF, tumor necrosis factor; IFN, interferon; IL, interleukin.

**Supplementary Table 5.** Levels of 11 cytokines in multipathogen-positive group and single pathogen-positive group

|  | **Multipathogen-positive**  **(n = 24)** | **Single pathogen-positive**  **(n = 11)** | ***P*** |
| --- | --- | --- | --- |
| TNF-α, pg/ml | 11.76 (5.23-84.73) | 18.73 (13.31-31.44) | 0.390 |
| IFN-γ, pg/ml | 54.07 (42.28-92.07) | 33.44 (20.91-56.62) | 0.061 |
| IL-2, pg/ml | 78.55 (51.92-111.97) | 64.38 (34.49-116.99) | 0.299 |
| IL-4, pg/ml | 32.95 (27.40-38.39) | 42.03 (19.18-55.04) | 0.472 |
| IL-5, pg/ml | 1.92 (1.59-3.56) | 2.03 (1.59-6.67) | 0.268 |
| IL-25, pg/ml | 124.91 (86.36-151.74) | 154.38 (83.87-464.08) | 0.252 |
| IL-6, pg/ml | 81.20 (7.34-273.20) | 14.05 (9.01-22.97) | 0.211 |
| IL-17A, pg/ml | 8.42 (4.49-17.77) | 20.70 (5.28-33.39) | 0.152 |
| IL-22, pg/ml | 19.19 (6.21-13.04) | 16.52 (8.70-36.64) | 0.067 |
| IL-23, pg/ml | 175.48 (93.80-245.87) | 210.51 (141.01-352.57) | 0.334 |
| IL-10, pg/ml | 5.67 (2.22-30.50) | 4.24 (0.71-10.49) | 0.430 |

**Abbreviations:** TNF, tumor necrosis factor; IFN, interferon; IL, interleukin.

**Supplementary Table 6.** Levels of 11 cytokines in other respiratory viruses-positive group and other respiratory viruses-negative group

|  | **Other respiratory viruses-positive**  **(n = 13)** | **Other respiratory viruses-negative**  **(n = 22)** | ***P*** |
| --- | --- | --- | --- |
| TNF-α, pg/ml | 9.91 (6.20-32.11) | 17.64 (5.81-48.18) | 0.555 |
| IFN-γ, pg/ml | 63.97 (38.91-128.08) | 44.48 (19.11-57.13) | 0.041 |
| IL-2, pg/ml | 89.54 (59.30-120.47) | 67.26 (39.10-97.80) | 0.229 |
| IL-4, pg/ml | 36.58 (28.03-42.68) | 31.12 (20.14-43.02) | 0.408 |
| IL-5, pg/ml | 1.81 (1.44-3.89) | 2.03 (1.59-6.67) | 0.302 |
| IL-25, pg/ml | 113.16 (83.87-133.39) | 138.60 (91.33-258.59) | 0.229 |
| IL-6, pg/ml | 98.02 (7.65-1155.33) | 21.06 (8.39-104.92) | 0.319 |
| IL-17A, pg/ml | 7.95 (4.79-10.33) | 14.36 (4.88-32.55) | 0.335 |
| IL-22, pg/ml | 8.22 (3.32-10.37) | 14.02 (9.12-31.66) | 0.026 |
| IL-23, pg/ml | 187.10 (100.93-253.75) | 175.48 (87.67-334.78) | 0.827 |
| IL-10, pg/ml | 9.74 (3.39-61.52) | 3.89 (0.71-10.07) | 0.049 |

**Abbreviations:** TNF, tumor necrosis factor; IFN, interferon; IL, interleukin.

**Supplementary Table 7.** Levels of 11 cytokines in different subgroups of patients

|  | **Bacteria-positive**  **(n = 13)** | **Bacteria-negative**  **(n = 22)** | ***P*** | **Fungi positive**  **(n = 6)** | **Fungi negative**  **(n = 29)** | ***P*** |
| --- | --- | --- | --- | --- | --- | --- |
| TNF-α, pg/ml | 16.12 (4.94-103.26) | 16.34 (6.46-29.63) | 1.000 | 6.94 (5.43-97.67) | 18.51 (6.14-41.08) | 0.379 |
| IFN-γ, pg/ml | 50.84 (28.46-105.48) | 47.70 (25.89-78.32) | 1.000 | 61.32 (45.72-313.60) | 45.73 (25.33-69.36) | 0.187 |
| IL-2, pg/ml | 78.55 (40.60-120.65) | 68.69 (53.41-107.64) | 0.801 | 95.30 (56.73-111.79) | 70.11 (46.70-114.32) | 0.480 |
| IL-4, pg/ml | 31.73 (28.03-37.78) | 35.38 (21.42-45.99) | 0.674 | 36.61 (33.40-40.73) | 30.50 (21.10-44.01) | 0.334 |
| IL-5, pg/ml | 2.03 (1.59-3.34) | 2.03 (1.59-4.66) | 0.801 | 1.59 (1.26-3.27) | 2.03 (1.59-4.10) | 0.134 |
| IL-25, pg/ml | 133.39 (88.84-205.01) | 124.91 (83.87-187.22) | 0.749 | 124.91 (105.84-159.79) | 133.39 (83.87-238.25) | 0.949 |
| IL-6, pg/ml | 47.45 (6.62-200.88) | 21.06 (8.52-146.47) | 0.960 | 198.64 (48.63-2212.25) | 19.14 (7.65-104.98) | 0.084 |
| IL-17A, pg/ml | 11.81 (4.07-30.15) | 8.89 (5.02-27.68) | 0.906 | 6.49 (4.54-11.44) | 10.82 (5.15-32.83) | 0.235 |
| IL-22, pg/ml | 11.50 (4.98-28.15) | 10.37 (6.76-16.66) | 0.601 | 9.82 (0.71-11.92) | 11.50 (7.59-28.15) | 0.218 |
| IL-23, pg/ml | 171.61 (78.90-311.08) | 191.00 (103.28-266.58) | 0.555 | 171.63 (95.36-251.78) | 179.35 (94.60-311.05) | 0.717 |
| IL-10, pg/ml | 5.31 (1.46-11.62) | 6.42 (0.71-17.85) | 0.906 | 5.13 (0.71-244.45) | 6.03 (1.46-12.00) | 0.782 |

**Abbreviations:** TNF, tumor necrosis factor; IFN, interferon; IL, interleukin.

**Supplementary Table 8.** Distribution of pathogens in SP group and NSP group

|  | **NSP**  **(n = 25)** | **SP**  **(n = 10)** | ***P*** |
| --- | --- | --- | --- |
| Multipathogen-positive | 15 (60.0%) | 9 (90.0%) | 0.120 |
| Other respiratory viruses-positive | 6 (24.0%) | 7 (70.0%) | 0.020 |
| Bacteria-positive | 10 (40.0%) | 3 (30.0%) | 0.709 |
| Fungi-positive | 3 (12.0%) | 3 (30.0%) | 0.322 |

**Abbreviations:** NSP, non-severe pneumonia; SP, severe pneumonia.

**Supplementary Table 9.** ROC curve analysis of various indicators to predict severe influenza A pneumonia

|  | **Cutoff points** | **AUC** | **95% CI** | ***P*** | **Sensitivity (%)** | **Specificity (%)** |
| --- | --- | --- | --- | --- | --- | --- |
| IL-4/IL-17A | >4.97 | 0.912 | 0.766 - 0.981 | <0.001 | 80.00 | 92.00 |
| NLR | >6.57 | 0.892 | 0.716 - 0.960 | <0.001 | 80.00 | 88.00 |
| PaO_2_ / FiO_2_ | ≤110 | 0.760 | 0.586 – 0.888 | 0.006 | 50.00 | 96.00 |
| CURB-65 | >1 | 0.774 | 0.601 – 0.898 | <0.001 | 50.00 | 92.00 |
| PSI | >92 | 0.798 | 0.628 – 0.914 | <0.001 | 70.00 | 84.00 |

**Abbreviations:** AUC, area under curve; CI, confidence interval; IL, interleukin; NLR, neutrophil/lymphocyte ratio; CURB-65, confusion, urea, respiratory rate, blood pressure, and age ≥ 65 years old; PSI, pneumonia severity index.

**Supplementary Table 10.** ROC curve analysis of various indicators to predict IPPV in patients with influenza A pneumonia.

|  | **Cutoff**  **points** | **AUC** | **95% CI** | ***P*** | **Sensitivity (%)** | **Specificity (%)** |
| --- | --- | --- | --- | --- | --- | --- |
| IL-6/IL-17A | >21.65 | 0.987 | 0.877 - 1.000 | <0.001 | 88.89 | 100.00 |
| IL-6/TNF-α | >3.63 | 0.983 | 0.869 - 1.000 | <0.001 | 100.00 | 92.31 |
| IL-10/IL-22 | >0.71 | 0.944 | 0.811 – 0.993 | <0.001 | 100.00 | 84.62 |
| IL-6, pg/ml | >127.44 | 0.923 | 0.781 – 0.986 | <0.001 | 77.78 | 96.15 |
| IFN-γ/IL-5 | >24.76 | 0.908 | 0.761 – 0.979 | <0.001 | 88.89 | 80.77 |

**Abbreviations:** AUC, area under curve; CI, confidence interval; IL, interleukin; TNF, tumor necrosis factor; IFN, interferon.

**References**

1. Niederman MS, Mandell LA, Anzueto A, Bass JB, Broughton WA, Campbell GD, et al. Guidelines for the management of adults with community-acquired pneumonia. Diagnosis, assessment of severity, antimicrobial therapy, and prevention. *Am J Respir Crit Care Med* (2001) 163(7):1730-54. Epub 2001/06/13. doi: 10.1164/ajrccm.163.7.at1010. PubMed PMID: 11401897.

2. Mandell LA, Wunderink RG, Anzueto A, Bartlett JG, Campbell GD, Dean NC, et al. Infectious Diseases Society of America/American Thoracic Society consensus guidelines on the management of community-acquired pneumonia in adults. *Clin Infect Dis* (2007) 44 Suppl 2:S27-72. Epub 2007/02/06. doi: 10.1086/511159. PubMed PMID: 17278083; PubMed Central PMCID: PMCPMC7107997.

3. Salih W, Schembri S, Chalmers JD. Simplification of the IDSA/ATS criteria for severe CAP using meta-analysis and observational data. *Eur Respir J* (2014) 43(3):842-51. Epub 2013/10/12. doi: 10.1183/09031936.00089513. PubMed PMID: 24114960.
